# Cross-species transcriptomics identifies obesity associated genes between human and mouse studies

**DOI:** 10.1186/s12967-024-05414-1

**Published:** 2024-06-25

**Authors:** Animesh Acharjee, Susanne N. Wijesinghe, Dominic Russ, Georgios Gkoutos, Simon W. Jones

**Affiliations:** 1 Institute of Cancer and Genomic Sciences, University of Birmingham, Birmingham, B15 2TT UK; 2 MRC Health Data Research UK (HDR UK), Birmingham, UK; 3 Institute of Translational Medicine, Foundation Trust, University Hospitals Birmingham NHS, Birmingham, B15 2TT UK; 4 Centre for Health Data Research, University of Birmingham, Birmingham, B15 2TT UK; 5 Institute of Inflammation and Ageing, MRC Versus Arthritis Centre for Musculoskeletal Ageing Research, University of Birmingham, Birmingham, UK

**Keywords:** Multi omics, Transcriptomics, Translational medicine, Obesity

## Abstract

**Background:**

Fundamentally defined by an imbalance in energy consumption and energy expenditure, obesity is a significant risk factor of several musculoskeletal conditions including osteoarthritis (OA). High-fat diets and sedentary lifestyle leads to increased adiposity resulting in systemic inflammation due to the endocrine properties of adipose tissue producing inflammatory cytokines and adipokines. We previously showed serum levels of specific adipokines are associated with biomarkers of bone remodelling and cartilage volume loss in knee OA patients. Whilst more recently we find the metabolic consequence of obesity drives the enrichment of pro-inflammatory fibroblast subsets within joint synovial tissues in obese individuals compared to those of BMI defined ‘health weight’. As such this present study identifies obesity-associated genes in OA joint tissues which are conserved across species and conditions.

**Methods:**

The study utilised 6 publicly available bulk and single-cell transcriptomic datasets from human and mice studies downloaded from Gene Expression Omnibus (GEO). Machine learning models were employed to model and statistically test datasets for conserved gene expression profiles. Identified genes were validated in OA tissues from obese and healthy weight individuals using quantitative PCR method (N = 38). Obese and healthy-weight patients were categorised by BMI > 30 and BMI between 18 and 24.9 respectively. Informed consent was obtained from all study participants who were scheduled to undergo elective arthroplasty.

**Results:**

Principal component analysis (PCA) was used to investigate the variations between classes of mouse and human data which confirmed variation between obese and healthy populations. Differential gene expression analysis filtered on adjusted p-values of p < 0.05, identified differentially expressed genes (DEGs) in mouse and human datasets. DEGs were analysed further using area under curve (AUC) which identified 12 genes. Pathway enrichment analysis suggests these genes were involved in the biosynthesis and elongation of fatty acids and the transport, oxidation, and catabolic processing of lipids. qPCR validation found the majority of genes showed a tendency to be upregulated in joint tissues from obese participants. Three validated genes, IGFBP2 (p = 0.0363), DOK6 (0.0451) and CASP1 (0.0412) were found to be significantly different in obese joint tissues compared to lean-weight joint tissues.

**Conclusions:**

The present study has employed machine learning models across several published obesity datasets to identify obesity-associated genes which are validated in joint tissues from OA. These results suggest obesity-associated genes are conserved across conditions and may be fundamental in accelerating disease in obese individuals. Whilst further validations and additional conditions remain to be tested in this model, identifying obesity-associated genes in this way may serve as a global aid for patient stratification giving rise to the potential of targeted therapeutic interventions in such patient subpopulations.

**Supplementary Information:**

The online version contains supplementary material available at 10.1186/s12967-024-05414-1.

## Introduction

The World Health Organisation (WHO) reports a significant increase in global obesity rates, with prevalence having nearly quadrupled since 1975, and projected to further increase in the foreseeable future such that by 2030, around 50% of adults in the United States will be afflicted with obesity, and 25% with severe obesity [1, 2]. The highest prevalence of obesity is reported in North America, Europe, and the Middle East, whereas East Asia demonstrates comparatively lower rates. The exponential rise in incidence of obesity over the past few decades has led WHO to officially acknowledge it as a global epidemic since 1997 [3]. Obesity is defined as a condition marked by an imbalance in the equilibrium between the amount of energy consumed and the amount of energy expended [4–6]. The escalation in the prevalence of obesity can be ascribed to a confluence of factors, namely heightened caloric consumption, and diminished levels of physical activity. High-fat diets [7–10] encourage the development of hypertension and glucose intolerance, as well as raised body adiposity and leptin levels. The resulting accumulation of saturated or long-chain fatty acids can lead to increased build-up of body fat through resynthesis of new triglycerides and ectopic deposition in other tissues which in turn increases the production of inflammatory cytokines and adipokines [11–13]. Indeed, the secretion of hormones, cytokines, and growth factors by adipose tissue is integral to the regulation of energy balance, and impacts on distal tissues such as skeletal muscle, articular joint tissues, heart and the liver. Consequently, obesity is closely linked to the development of several serious ailments, including type 2 diabetes, cardiovascular disease, cancer, inflammatory joint disorders, and liver disease.

Multiple studies have investigated the effect of several factors which influence obesity, including diet [7], inflammation [8, 9], microbiome [10, 11] and lifestyle [12]. These have utilised mouse models (including transgenics) and human tissues to understand the pathobiology and pathophysiology related to obesity. To this end, transcriptomic approaches such as bulk and single-cell RNA-sequencing (RNA-seq) [14] are proving to be highly valuable techniques for investigating gene expression patterns across diverse species, tissues, and cell populations, yielding important insights into the genetic implications of the progression of obesity with the potential to identify novel druggable targets for combating obesity and obesity-associated co-morbidities. Wijesinghe et al. [15], investigated the effects of obesity in OA disease pathogenesis utilising multi-omic approaches to phenotype synovial joint tissue in patients afflicted with OA, in joints which were either weight-bearing (e.g. hips, knees) or non-weight bearing (e.g. hand joints). This study determined that OA synovial fibroblasts can be characterised by distinct molecular endotypes affected by obesity, joint stress, and anatomical site, and are differentially distributed between OA patients who are either obese or of lean weight [15].

In another study, Rey et al. [16], profiled obesity-associated non-coding RNAs (lncRNAs) in subcutaneous adipose tissue by bulk RNA-seq analysis. The study identified a total of 171 differentially expressed genes, including 11 ncRNAs which pathway enrichment analysis correlated to underlying biological mechanisms involved in adipocyte differentiation, insulin response, immune cell activation and fatty acid metabolism. Corno et al. [17], investigated the effects of body mass index (BMI) and polyunsaturated fatty acids on tumorigenesis by transcriptomic profiling of human visceral adipocytes taken from patients with obesity and colorectal cancer. Gene ontology and pathway enrichment analysis identified DEGs involved in fibrosis, inflammation and metabolism of pyruvate, glucose and lipids. Nasias et al. [18] conducted a comprehensive analysis of gene expression changes in white adipose tissue during diet induced MetS in mice [18]. The results indicated a wide range of differentially expressed genes, highlighted key pathways related to metabolism and immunity, and pointed towards the involvement of cytokines in the development of MetS. [18]. Another RNA-seq mouse experiment performed by Cao et al. 2018 found differential gene expression in brown adipose tissue of lean and obese mice after four weeks of high-fat diet, suggesting its potential role in obesity, insulin resistance, and inflammation [19].

This present study employed a number of public gene expression datasets from human and mice pertaining to obesity in an effort to ascertain genes that have the patient stratification and therapeutic potential. In addition to this, we have identified common obesity-associated genes across species and tissue types, which are likely central mediators in obesity related diseases, and validated those genes by quantitative PCR method in human synovial joint tissues.

## Methods

### Study recruitment

UK National Research Ethics Committee (NRES 16/SS/0172) granted ethical approval for post-operative collection of joint tissue from consenting patients with OA. Study recruitment was coordinated by research nurses at the Royal Orthopaedic Hospital, Birmingham (United Kingdom) and Russell’s Hall Hospital, Dudley (United Kingdom).

### Differential gene expression analysis

Genes are commonly assessed according to their fold change, which represents the extent of expression variations, and by their respective p-values, which measure the statistical significance of such variations. Genes with significant fold changes (fold changes ± 1.5) and low p-values (p < 0.05) are considered statistically significant differentially expressed. Multiple testing correction was performed to control for false positives that may arise due to testing multiple genes simultaneously, and multiple testing correction methods (BH false discovery rate correction) were applied to adjust p-values (adj p < 0.05).

### Datasets

All the datasets were obtained from Gene Expression Omnibus (GEO) on 29th October 2022. Those data sets are from both human and mouse samples. A detailed summary of each dataset can be found in Table 1.

**Table 1:** List of the public data sets, organism and tissue sources used in this study

GEO data	Organism	Type of dataset	Number of participants			Tissue source
			Lean	Obese	Total	
GSE24883	Human	Array	32	32	64	Subcutaneous and visceral adipose tissue
GSE59034	Human	Array	16	16	32	Subcutaneous and visceral adipose tissue
GSE49195	Mouse	Array	6	6	12	Lean and obese Liver
GSE39375	Mouse	Array	5	5	10	Lean and obese Liver
GSE152815	Human	Single Cell RNA sequencing	4	4	8	Hand, hip, knee, and foot Joints
GSE219027	Human	Bulk RNA sequencing	24	24	48	Hand, hip, knee, and foot Joints

### Patients and tissue samples

Obese and lean-weight patients, categorised by BMI > 30 and BMI between 18 and 24.9 respectively, were recruited for the study. These patients were scheduled to undergo elective arthroplasty for OA and synovium was collected from hip or knee joints (N = 38). Ethics approval was provided by the UK National Research Ethics Committee (approval no. 14/ES/1044), and informed consent was obtained from all patients. The characteristics of study participants are summarised in Table 2.

**Table 2:** Summary of participant characteristics. Data displayed as mean ± standard deviation

	Lean N = 16	Obese N = 22	P-value
Age in years	69.1 ± 2.6	66.7 ± 1.8	0.4382
Gender (Number of female participats, (%))	12 (75%)	12 (55%)	0.2071
Hip: Knee Ratio (Number of hip joints, (%))	12 (75%)	12 (55%)	0.0717
BMI	22.8 ± 0.4	35.9 ± 1.3	< 0.0001
Waist: Hip Ratio	0.8 ± 0.02	0.9 ± 0.01	0.0007

*N* Number of participants

### Quantitative real-time PCR validations of identified genes

Total RNA was isolated from synovium using TRIzol (Life Technologies). Primers for individual transcripts of interest (see Supplementary Table 1) were either predesigned TaqMan™ Gene Expression Assay (FAM) from Life Technologies Ltd or designed using NCBI’s Primer-BLAST tool and Easy Oligos were ordered from Merck Life Science UK Ltd. PCR was performed from total RNA in a one-step reaction using either iTaq Universal One-Step or iTaq™ Universal SYBR® Green One-Step Kit from BioRad. Relative expression was determined using the ΔΔCt method, followed by normalization of values to those of 18S and GAPDH.

### Statistical and machine learning methods

Multiple statistical and machine learning models were used to identify genes differentiating between obesity and lean samples (DEG summary in Supplementary Table 1). Unsupervised Principal Component Analysis (PCA), a dimensionality reduction technique, was used in machine learning and data analysis. PCA seeks to preserve the underlying structure and relationships in the data while reducing its dimensionality, making it easier to visualize and analyse. Logistic regression was used to evaluate how well different classes could be distinguished. To estimate sample size, an inhouse developed tool known as Tools was used [20]. qPCR data was plotted using GraphPad PRISM with unpaired student’s t-test.

### Pathway enrichment analysis

**Fig. 1:**
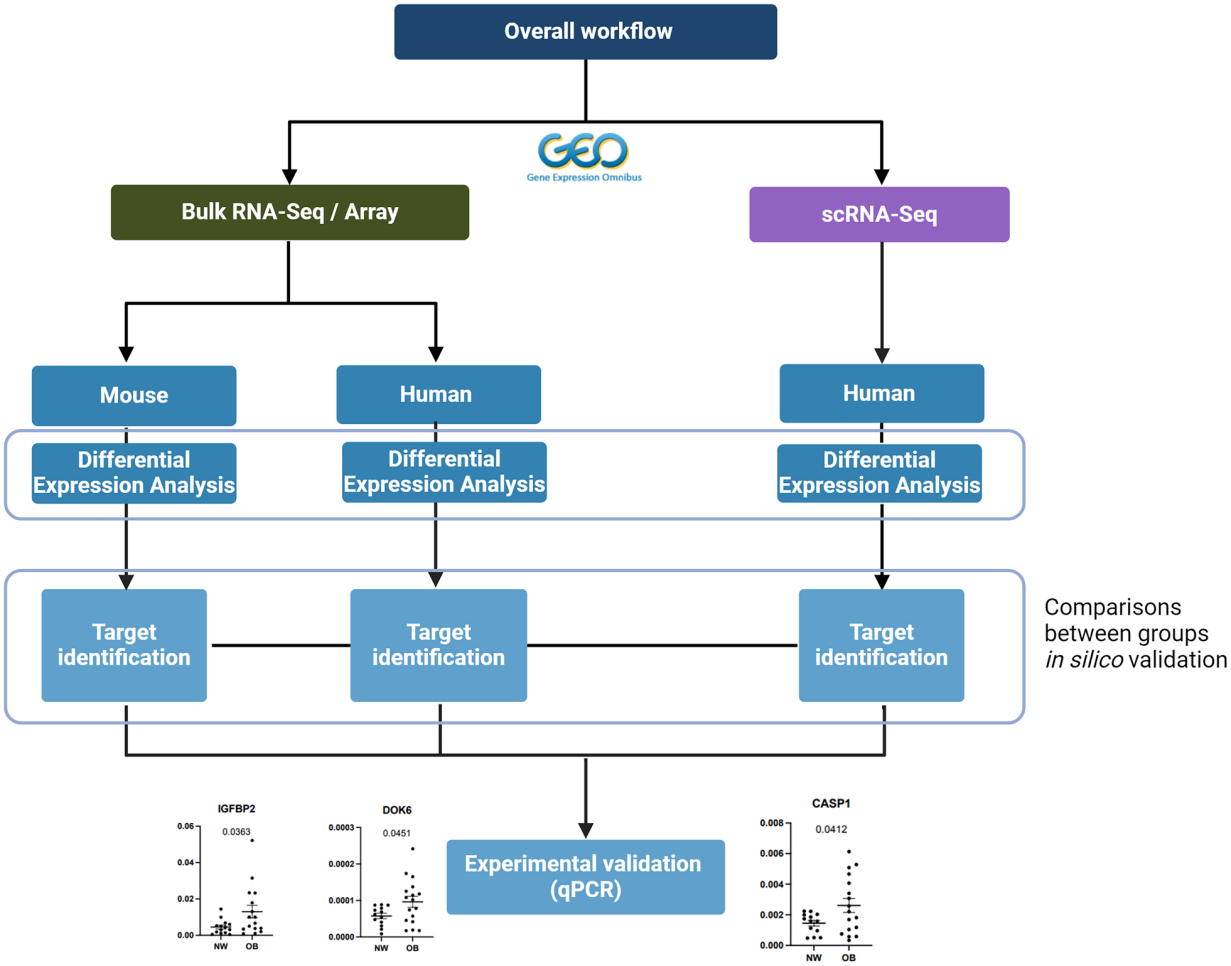
Overview of the gene target identification workflow using public and internal RNA sequencing data

Pathway enrichment analysis was performed using the Enrichr tool to determine functional mechanisms informed by differentially expressed genes derived from the datasets analysed. This approach identifies biological pathways that show enrichment in a gene list beyond what would be expected by random chance. We have used ShinyGO [21] and Enrichr [22] for this analysis. The analysis workflow is illustrated in Fig. 1.

## Results

### Mouse data analysis

**Fig. 2:**
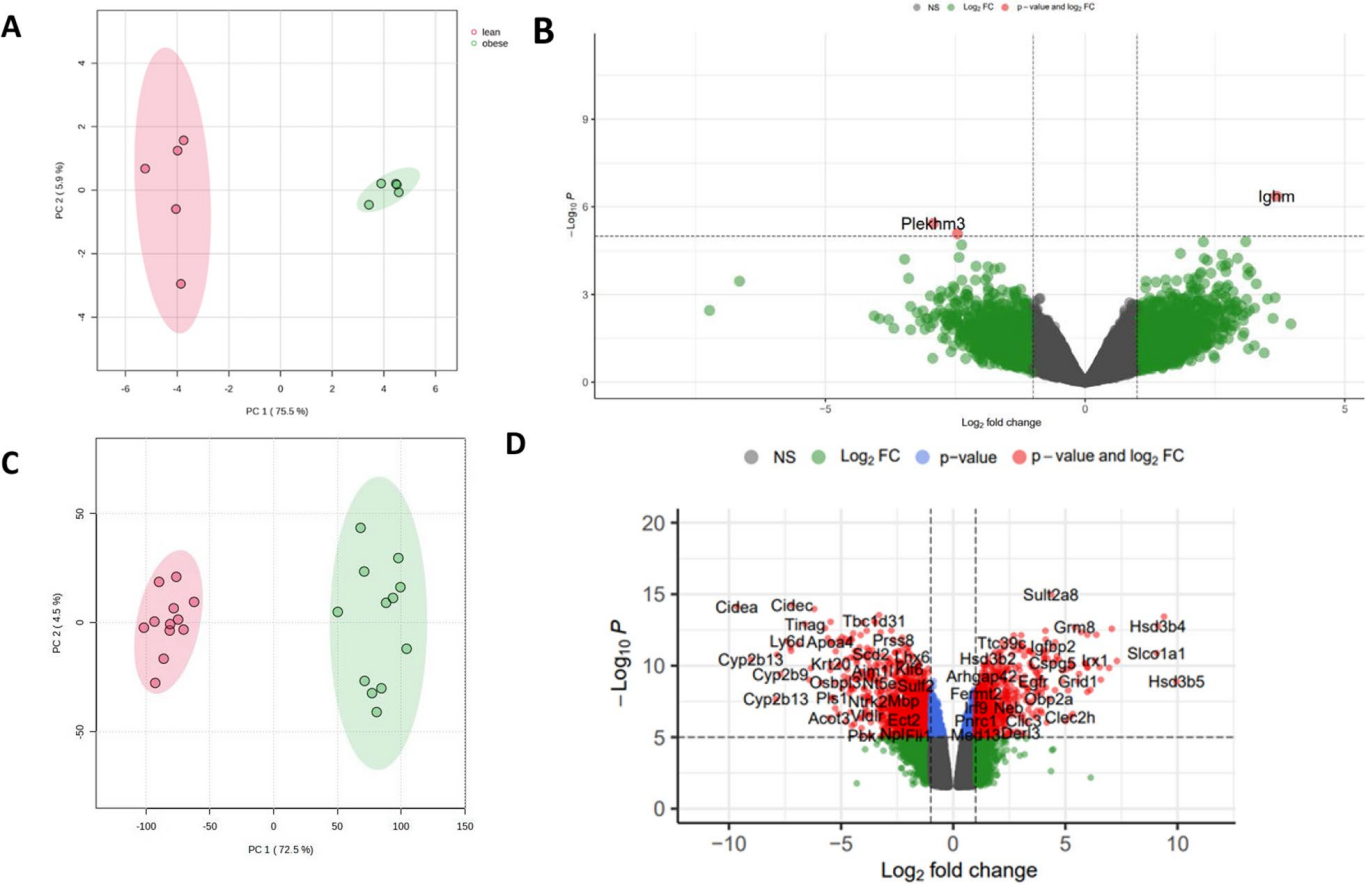
Differential gene expression analysis of mouse datasets GSE39375 and GSE49195. **A** PCA of GSE39375 data used to visualised lean vs. obese samples from mice. **B** Volcano plots used to visualise up and down regulated genes across lean and obese samples for GSE39375 dataset. **C** PCA of GSE49195 data used to visualise the lean vs. obese samples from mice. **D** Volcano plots used to visualise up and down regulated genes across lean and obese samples for GSE49195 dataset

Two mouse datasets were used GSE39375 and GSE49195 to identify differentially expressed influenced by obesity (summarized in Table 1). PCA was used to investigate the variations across two classes of mice which showed clear variation and separation between lean and obese mice in datasets GSE39375 and GSE49195, respectively (Fig. 2A and C). In dataset GSE39375, using an adjusted p value threshold of 0.2, twelve differentially expressed genes were identified where five genes were significantly upregulated, and seven genes were significantly downregulated in obese mice liver tissues compared to lean mice liver tissues (N = 6, 3 replicates per group) (Fig. 2B). Similar, differential gene expression analysis of GSE49195 identified 13,581 significantly differentially expressed genes (p adjusted, p < 0.05) of which 5,996 genes were downregulated and 7,585 were upregulated (Fig. 2D).

### Human data analysis

**Fig. 3:**
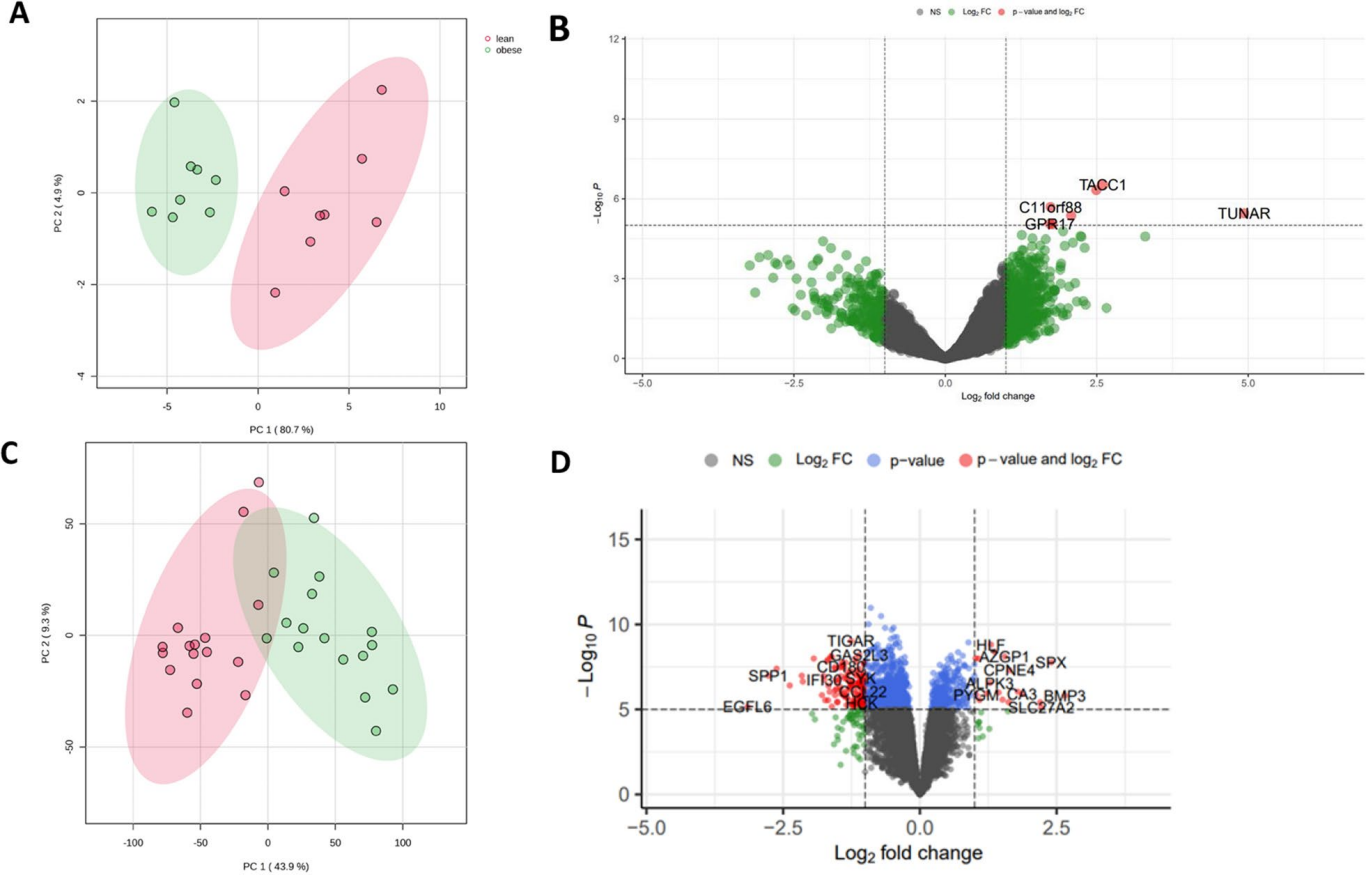
Differential gene expression analysis of public human datasets GSE24883 and GSE59034. **A** PCA for the GSE24883 used to visualise lean vs. obese patient adipose tissue samples **B** Volcano plots used to visualise up and down regulated genes across lean and obese samples for GSE24883 dataset. **C** PCA for the GSE59034 used to visualise the lean vs. obese samples **D** Volcano plots used to visualise up and down regulated genes across lean and obese samples for GSE59034 dataset

Three human datasets, namely GSE24883, GSE59034 and GSE219027 were analysed to identify potential targets which differentiate obese and lean populations. PCA analysis shows the variations across the dataset from GSE24883 and identified 24 genes of which two genes were downregulated and 22 genes were upregulated in obese subcutaneous and visceral adipose tissues compared to lean tissues (Fig. 3A and B). In a similar dataset, GSE59034, PCA analysis confirmed data separation although arguably considerably less than other datasets. In total, 7,127 differential expressed genes were identified, of which 3540 genes were upregulated and 3,587 genes were downregulated in obese subcutaneous and visceral adipose tissues compared to lean tissues (Fig. 3C and D).

**Fig. 4:**
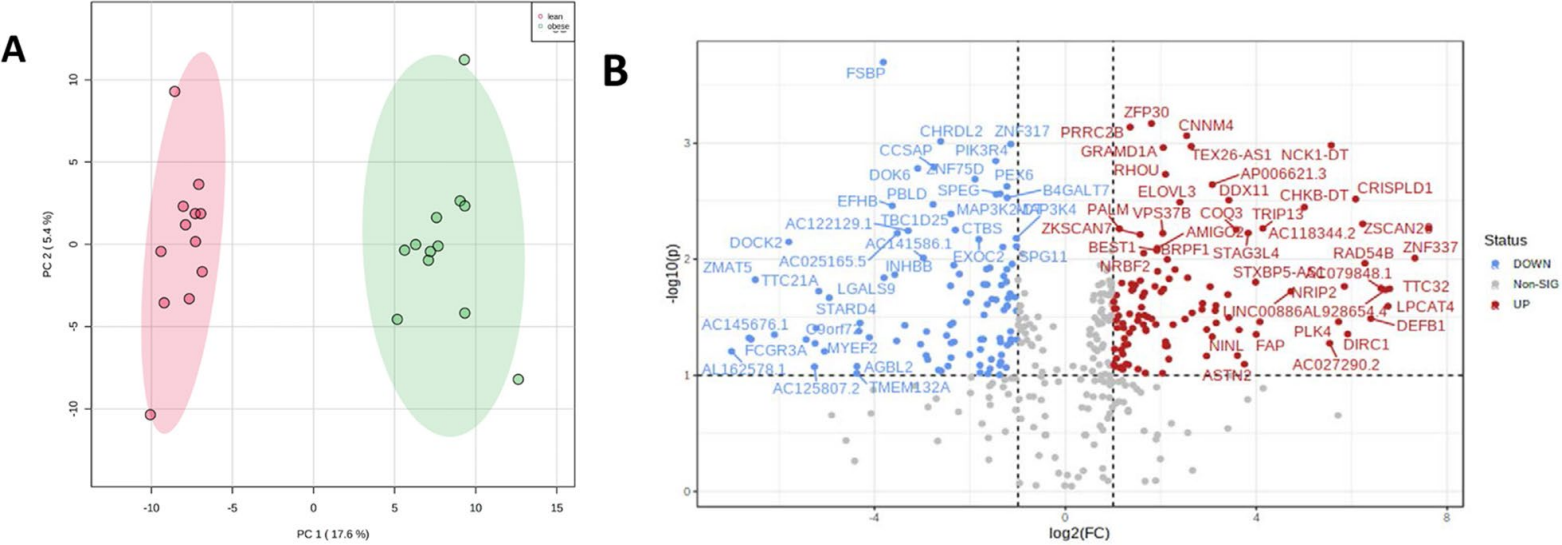
Differential gene expression analysis of internal human data set GSE219027. **A** PCA score plot showing the differences between obese and lean patient joint tissue samples. Red and green ellipse indicates confidence interval of the patient cohorts **B** Volcano plots used to visualise up and down regulated genes across lean and obese patient joint tissue samples

The in-house dataset previously published by Wijesinghe et al. [15], GSE219027, PCA analysis showed a clear separation between obese and lean groups (Fig. 4A), Here, a total of 416 differentially expressed genes were identified, of which, 185 were upregulated and 231 were down regulated in obese synovial joint tissues compared to synovial joint tissues from lean populations (Fig. 4B). Differentially expressed genes were then used to identify a subset of key genes associated with obesity. Using iterative t-test, area under curve (AUC) values were estimated on selected genes with p < 0.01. This resulted in a final 12 genes used for logistic regression analysis yielding AUC of 0.98. This analysis was repeated in the other human datasets, GSE59034 and GSE24883 which resulted in AUC of 0.96 and 0.54, respectively. Similarly for mouse datasets, GSE39375 and GSE49195, AUC estimates were 0.63 and 0.81, respectively (Supplementary Fig. 1).

### Single cell RNA-sequencing vs. bulk RNA-sequence data

In the previously published study by Wijesinghe et al. [15] scRNA-seq of synovial joint fibroblasts from OA patients with either lean or obese BMI identified 8 synovial fibroblast clusters (GSE152815). Comprised of novel gene signatures, these 8 populations were identified to be differentially distributed where individuals with an obese BMI presented with molecular signatures reminiscent of pro-inflammatory fibroblasts known to drive disease pathogenesis. Gene lists and full data analysis pipelines are reported in the original publication which includes gene lists. Here we utilised the published single-cell fibroblast gene signatures and compared these against those genes identified in synovial tissues of obese populations by bulk RNA-seq analysis described above (GSE219027). This analysis identified the most frequently overlapping genes between scRNA-seq clusters and bulk RNA-seq datasets were PTN, UBE2S, NMB, PALM, SLC7A8, BTG2, MET, PAMR1, PTGS2, FABP3, PPIB and CDKN3 (Supplementary Fig. 2).

### Biological significance of the identified genes

The significant differentially expressed genes from mouse datasets, GSE49195 and GSE39375, were used to perform a pathway-based enrichment analysis which identified several metabolism-related pathways, including fatty acid elongation, biosynthesis of unsaturated fatty acid, AMPK signalling pathways (p < 0.05). Similarly, a pathway-based enrichment analysis using the significant differentially expressed genes from human datasets, GSE24883 and GSE59034, identified pathways such as lipid transport, lipid oxidation, lipid localization, long chain fatty acid transport, and lipid catabolic process (p < 0.05) (Supplementary Fig. 3).

### Design and power study of the identified genes

**Fig. 5:**
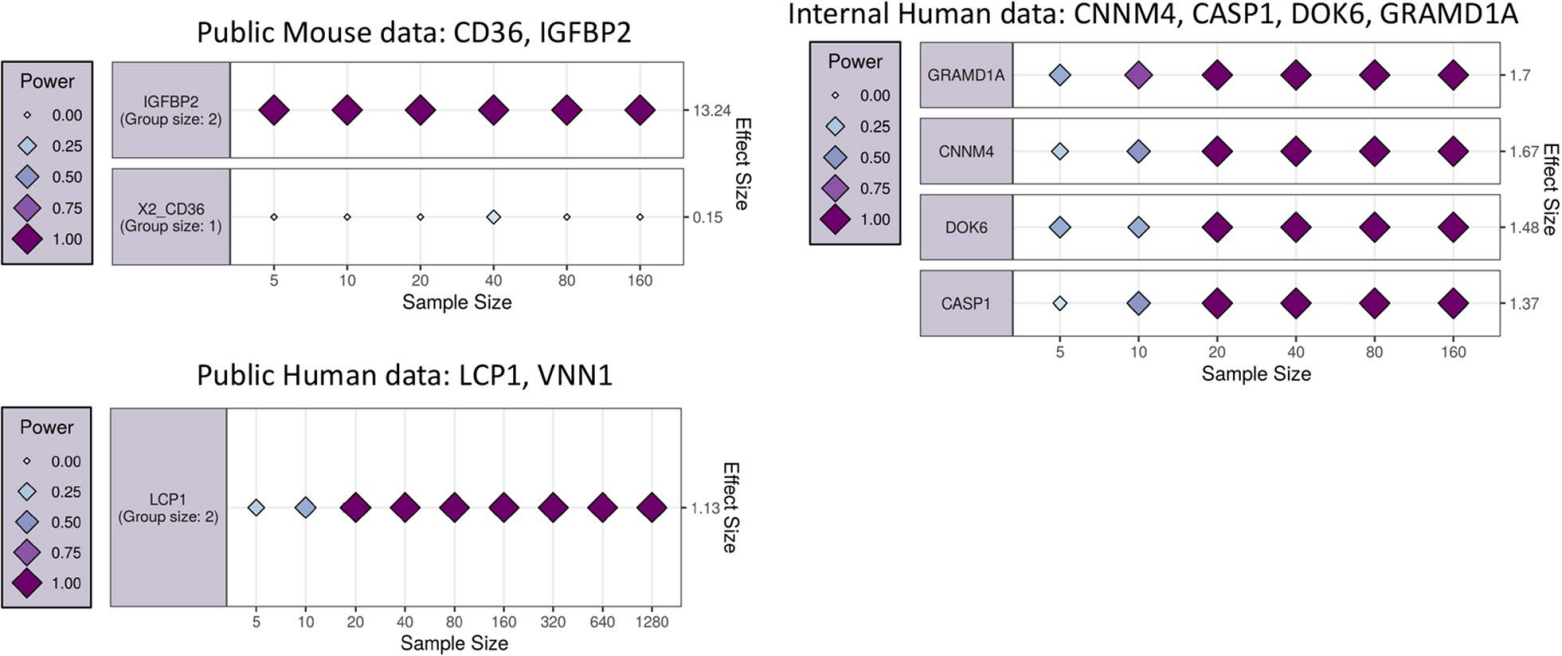
The two groups of correlated features identified by the power function are represented by the group member with the largest observed effect size. The effect size of each assessed variable is shown along the y-axis and a series of sample sizes along the x-axis. Power values determined for each effect/sample size combination using a simulated dataset with the same correlation structure as input data and displayed using variably sized/coloured rhombi

For each dataset, we selected relevant genes based on differential expression and biological significance. We have estimated the number of the genes required for future study and validation using PowerTools. Here, genes with similar expression were combined using Pearson based correlation and hence effect size was estimated (Fig. 5).

### qPCR validation

**Fig. 6:**
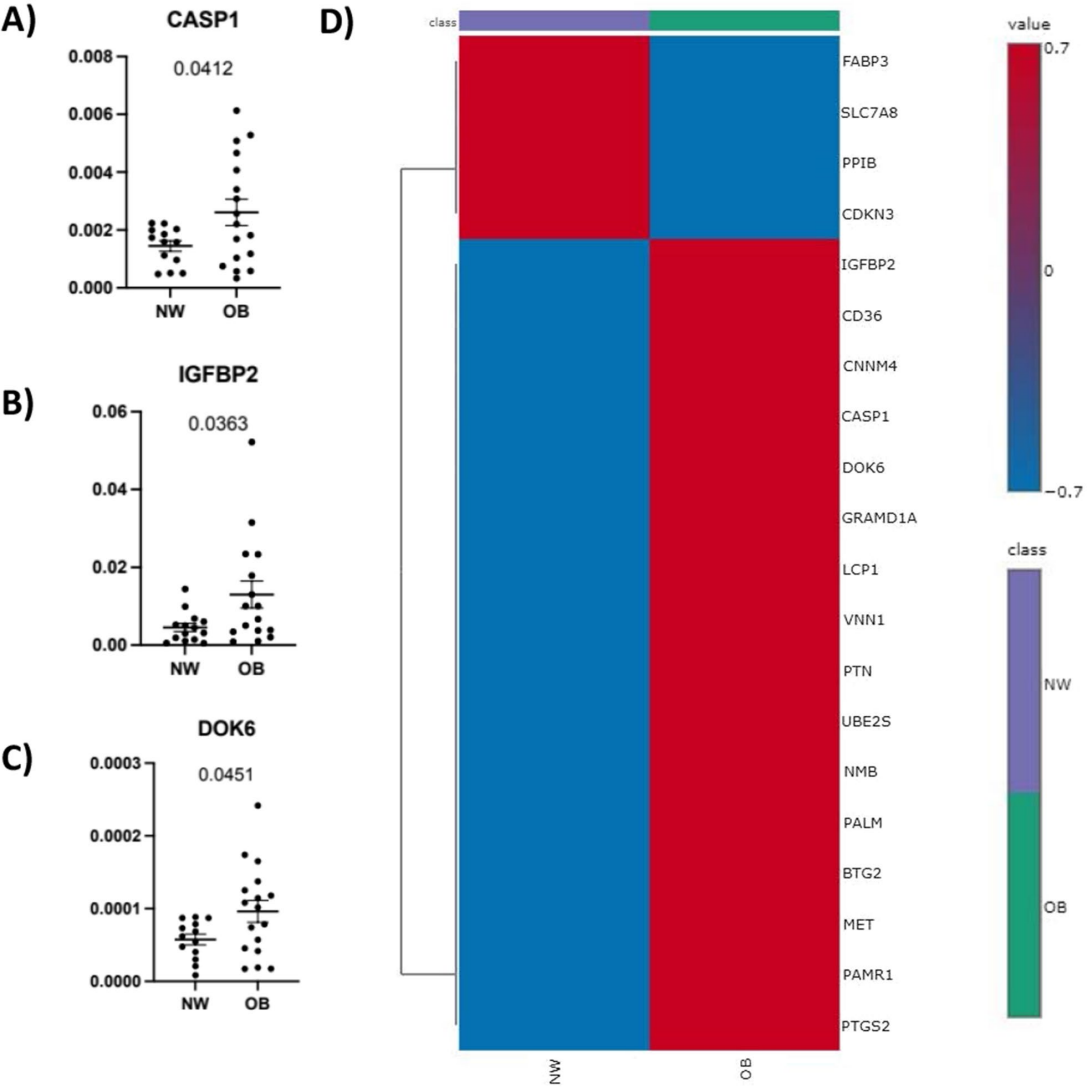
Validation of identified genes of interest in human tissue. Up and down regulated genes from computational analysis were validated using qPCR experiment **A** CASP1 **B** IGFBP2 **C** DOK6 are plotted, where each dot represents an individual patient, error bars represent standard error of the mean and p-value was calculated using unpaired student’s t-test. **D** Heatmap summarising average expression of the selected genes across lean (NW) and obese (OB) human synovial tissue samples. Red indicated high expression values and blue indicated lower expression. Individual plots can be found in Supplementary Fig. 3

From the genes identified from both the PowerTools analysis and overlap analysis, 20 genes in total were selected for validation using qPCR. Here, three of the identified genes (CASP1, IGFBP2 and DOK6) were statistically significantly upregulated (p < 0.05) in obese synovial tissues, compared to tissues from lean patients (Fig. 6A, B, C). Other genes showed a tendency towards upregulation in the obese samples where overall, four genes (SLC7A8, CDKN3, FABP3, PPIB) were downregulated in the obese samples and the remaining 16 genes were upregulated, compared to lean samples (Fig. 6D and Supplementary Fig. 4).

## Discussion

Obesity is highly heterogeneous and variable across species and tissue types influenced by a variety of factors including gender, race, ethnicity, age of onset, and genetics. As the prevalence of obesity rapidly increases there is a pressing need to identified obesity-associated targets which may have central roles in mediating obesity related diseases. In this study, human and mouse datasets were employed in a comprehensive transcriptomics endeavour to identify obesity-associated transcriptional signatures which were subsequently validated experimentally.

Our bioinformatics findings identified several obesity-associated genes across tissues, species, and patient cohorts. Throughout there are differences between the overall number of DEGs identified across mouse and human data as well as within species. This may be due to experimental design of the independent datasets including sample collection, handing and experimental procedure. There were also particular discrepancies in the number of samples used across studies, which no doubt contributed to variation. Additionally, these results may be impacted by different tissues used in each study, which have different functional relevance related to obesity versus disease. This likely holds true for LCP1 and VNN1m which were identified in published human datasets as downregulated in obese adipose tissues but upregulated in obese OA tissues. Investigating the AUC values in these datasets revealed that the model performance is low which is expected based on the analysis.

In mice datasets, we identified CD36, an obesity influenced gene that codes for a receptor of fatty acids [23], which was also detected in human datasets and in tissue validation although not significant. Another obesity-associated gene upregulated in mice data was IGFBP2, which was also confirmed in published human datasets. This was further validated by qPCR validation where we report for the first time that IGFBP2 is significantly upregulated in obese synovial tissues from OA patients. Interestingly, IGFBP2 is highly abundant in both OA and RA synovial fluid [24, 25]. IGFBPs are specifically elevated in sites of degenerated cartilage where they modulate the activity and bioavailability of IGF which is important for anabolic regulation in chondrocytes [26]. Our results here suggest that in obese individuals, IGFBP2 is produced at higher levels in synovium tissue, where it is known to maintain joint synovial fluid, and as such may facilitate cartilage anabolism in the damaged joint. Interestingly, IGFBP2-overexpressing transgenic mice are protected from both high-fat diet induced obesity and age-induced insulin resistance with improved glucose tolerance [27]. Further to this, elevated IGFBP2 in patients following bariatric procedures is associated with insulin sensitisation and in a rodent model IGFBP2 deficiency impairs the loss in adiposity and insulin sensitivity usually induced by such surgery [28]. This is likely due to IGFBP2 being under the regulation of leptin, a hormone linked to appetite suppression and maintenance of healthy body weight [29]. However, IGFBP2 is also a ligand for several targets capable of regulating inflammation associated signalling pathways in OA including PI3K/Akt/ MAPK/ PKC pathways [30, 31] whilst low IGFBP2 serum levels are associated with risk of metabolic syndromes [28] and implicated in glucose and lipid metabolism [31]. Additionally, studies report IGFBP2, through the STAT3 pathway, induces lung fibrosis and inflammation in rats with severe pneumonia [32]. In keeping with this, we have previously reported OA synovial fibroblasts from obese patients are highly inflammatory, proliferative, and metabolically dysregulated [33]. Coupled with our findings here, IGFBP2 upregulation in obese synovial tissue may explain the pro-inflammatory metabolic dysfunction in obese synovial fibroblasts which accelerates joint damage in obese patients [33], Whilst elevated IGFBP2 may be beneficial in certain models, it’s increase in the synovial micro-environment potentially drives disease pathogenesis. Further investigation will be necessary to determine the role of IGFBP2 in disease pathogenesis within joint synovium and its potential to resolve metabolic inflammation.

Several genes identified in our internal bulk and single RNA-seq data was also detected in the published human datasets analysed including CASP1, DOK6, PTN, SLC7A8, BTG2, PTGS2, CDKN3, FABP3 and PPIB. Whilst experimental validation in tissues showed a similar trend of gene expression as observed in published datasets, CASP1 and DOK6 were the only genes found to be significantly upregulated in the synovial joint tissues of obese individuals which we report here for the first time. CASP1 encodes the evolutionary conserved inflammatory mediator Caspase-1. Through the assembly of the inflammasome complex [34], caspase-1 activates pro-inflammatory cytokines IL1β and IL18 which are known to induce joint damage in OA through induction of inflammatory signalling pathways NF-κB and p38 MAPK kinase [35–38]. Additionally, pyroptosis, a caspase-1-dependent programmed cell death, is thought to be triggered by OA-related risk factors such as obesity-associated cholesterol thus promoting joint degradation which is likely accelerated in individuals with obesity. Pyroptotic synovial macrophages and synovial fibroblasts also contribute to synovitis and fibrosis, which further drive OA disease progression [36]. Interestingly, Caspase-1 is linked to adipocyte function and metabolic regulation including lipid metabolism and glucose homeostasis [38]. Inflammation-associated lipid metabolism is associated with metabolic disorders suggesting an association between Caspase-1 regulated lipid metabolism and obesity-associated inflammation [38, 39]. Indeed, caspase-1 deficient mice are protected from high-fat diet induced accumulation of circulating triglycerides, hepatic steatosis and inflammation [40, 41]. However, caspase-1 deficient mice have increased susceptibility to high-fat-diet-induced adiposity, with increased subcutaneous and total body adipose tissue volumes, promoting inflammatory macrophage infiltration in adipose tissues which in turn accelerates obesity-associated inflammation [42].

DOK6 is a novel marker reported here for the first time in OA synovium tissue which is elevated in joints of patients who are obese. Mouse studies have largely found DOK6 to be expressed within the central nervous system and involved in neurite outgrowth through positive regulation of the MAPK pathway [44, 45]. In humans DOK6 is reportedly expressed in immune cells, particularly induced in activated CD8 + T-cells, which are also in OA synovial tissues [46, 47]. Whilst little is known about the functional relevance of DOK6 in obesity, other members of the DOK family are cited as susceptibility genes for obesity and diabetes in North Indian population [48] and have been linked to adipocyte hypertrophy in high-fat diet fed mice [49] [50], which may be of relevance in OA where pain severity increases with obesity [51]. Indeed DOK6, will be an interesting marker to follow up in future OA studies with relevance of obesity-associated joint damage and inflammation. Of the genes identified in our internal bulk and single RNA-seq data only PTN, SLC7A8 and CDKN3 were also identified in mice datasets to be similarly expressed. PTN is a cytokine which when knocked out in high-fat diet fed mice, protects against insulin resistance, obesity and neuroinflammation [52]. Similarly, deletion of SLC7A8 in mice offers significant protection against diet-induced obesity and enhances glucose metabolism. The deficiency in SLC7A8 mitigates the enlargement of adipocytes in both white and brown adipose tissues and decreases lipid build-up in various organs [53]. CDKN3 gene expression has been found to be increased in obese individuals, which may be linked to modifications in cellular functions related to metabolism and adipogenesis. Genetic variations within CDKN3 have been identified as potential elements that can impact on the risk of developing obesity. Other genes such as BTG2, involved in suppressing the JAK2–Stat3 signalling pathway [54], PTGS2, expressed within adipose tissue [55], and FABP3 involved in the metabolism of fatty gene [56] are also candidate targets for follow up studies, with reported functions involved in lipid metabolism, persistent inflammation and irregular fat storage, respectively. These findings highlight the importance of understanding the role of these genes in the development of obesity and their potential impact on metabolism and adipogenesis.

The observations presented in this study should be considered in view of experimental limitations. First, greater sample numbers would have improved the statistical power of the study and reduced the risk to accumulate false positives. However, it is important to note that this is a caveat for many human tissue studies, particularly those where surgery is required for tissue collection. Second, the fold change cut off used in the differential gene expression analysis is based on previous studies. Further, it's important to note that genes that share expression patterns might not always perform comparable tasks in the pathways. And finally, post-transcriptional and other levels of control may not coordinate transcription patterns for genes with comparable functions. Nevertheless, the validated genes reveal significant potential for further targeted experiments and follow up clinical studies.

To conclude, the present study has identified obesity-associated genes, which we have validated and reported in obese arthritic joint tissues for the first time. Our findings suggest obesity-associated genes are conserved across conditions and may therefore be fundamental in accelerating disease in obese individuals. In particular, genes such as IGFBP2 and CASP1, with functions related to obesity and metabolism, may explain some of our previous findings of metabolically dysregulated fibroblasts in OA synovial tissue from obese patients [33]. Whilst further validations and additional conditions remain to be tested in this model, identifying obesity-associated genes using the approach we outline here may serve not only as a global aid for patient stratification but also give rise to the potential of targeted therapeutic interventions in specific patient subpopulations.

### Supplementary Information

Additional file 1: Figure 1: Venn diagram of the overlapping genes between bulk RNA sequence and single cell RNA sequence.

Additional file 2: Figure 2: Area under the curve using logistic regression on the selected genes across all the datasets.

Additional file 3: Figure 3: Pathway analysis of the genes identified from the data sets. Figure 3: Validation of identified genes of interest in human tissue. Plots of differentially expressed genes from computational analysis which were validated using qPCR, where each dot represents an individual patient, error bars represent standard error of the mean and p-value was calculated using unpaired student’s t-test. NW - lean and OB – obese.

Additional file 4: Figure 4: Validation of identified genes of interest in human tissue. Plots of differentially expressed genes from computational analysis which were validated using qPCR, where each dot represents an individual patient, error bars represent standard error of the mean and p-value was calculated using unpaired student’s t-test.

Additional file 5: Table 1: List of primers and assay reagents used for qPCR validations.

Additional file 6: Table 2: Summary of the differentially expressed genes (DEG) identified in expression datasets analysed.

## Data Availability

The datasets analysed during the current study are available in the Gene Expression Omnibus.
