# Supplementary figures and images for: Cross-species transcriptomics identifies obesity associated genes between human and mouse studies
Source: J Transl Med. 2024 Jun 25;22:592. doi: 10.1186/s12967-024-05414-1 (PMC11197204; doi:10.1186/s12967-024-05414-1)

## Slide 1
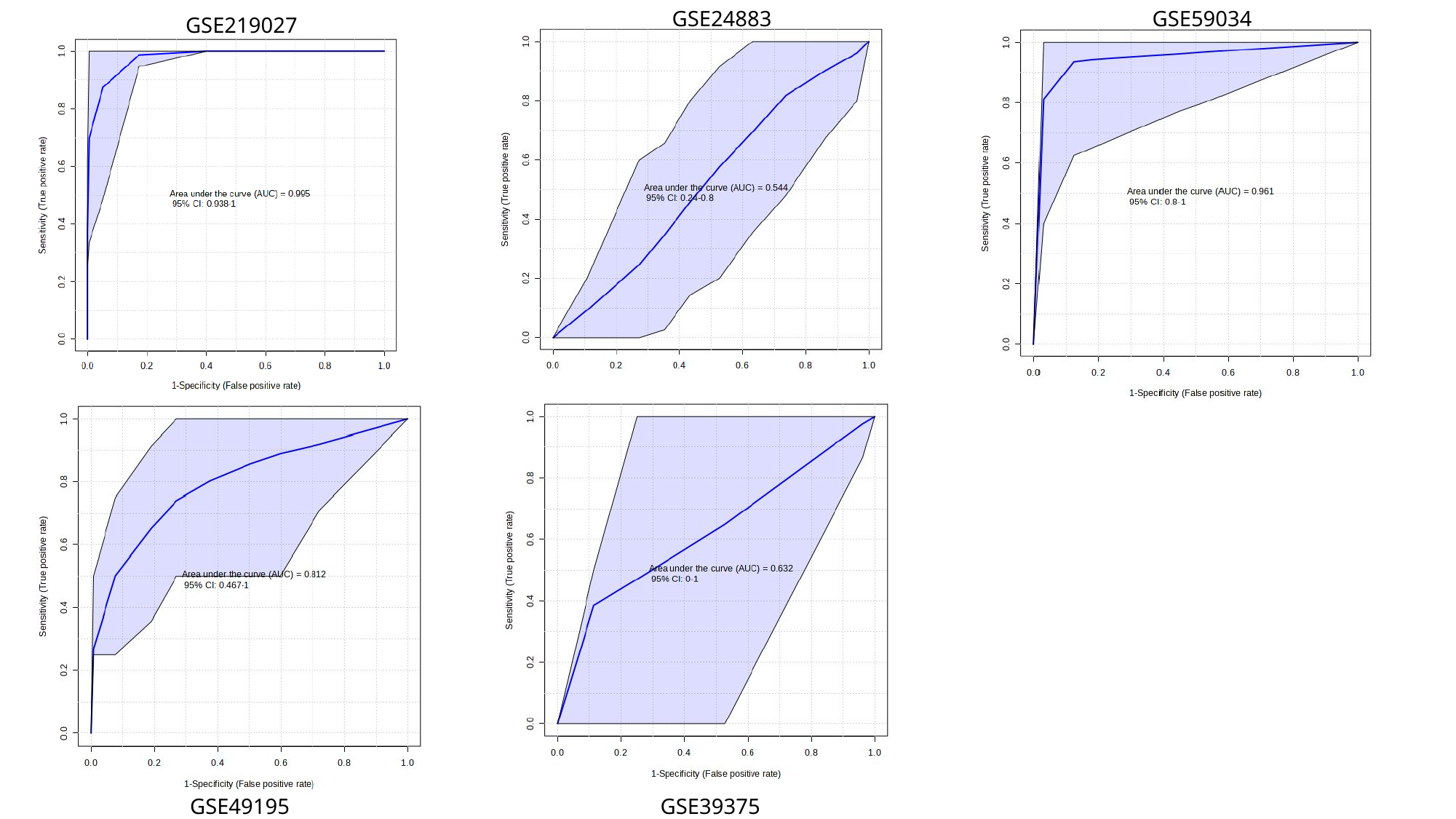

GSE24883
GSE59034
GSE219027
GSE49195
GSE39375

Supplement: Supplementary file 1 — Additional file 1: Figure 1: Venn diagram of the overlapping genes between bulk RNA sequence and single cell RNA sequence. [file 12967_2024_5414_MOESM1_ESM.pptx]

## Slide 1
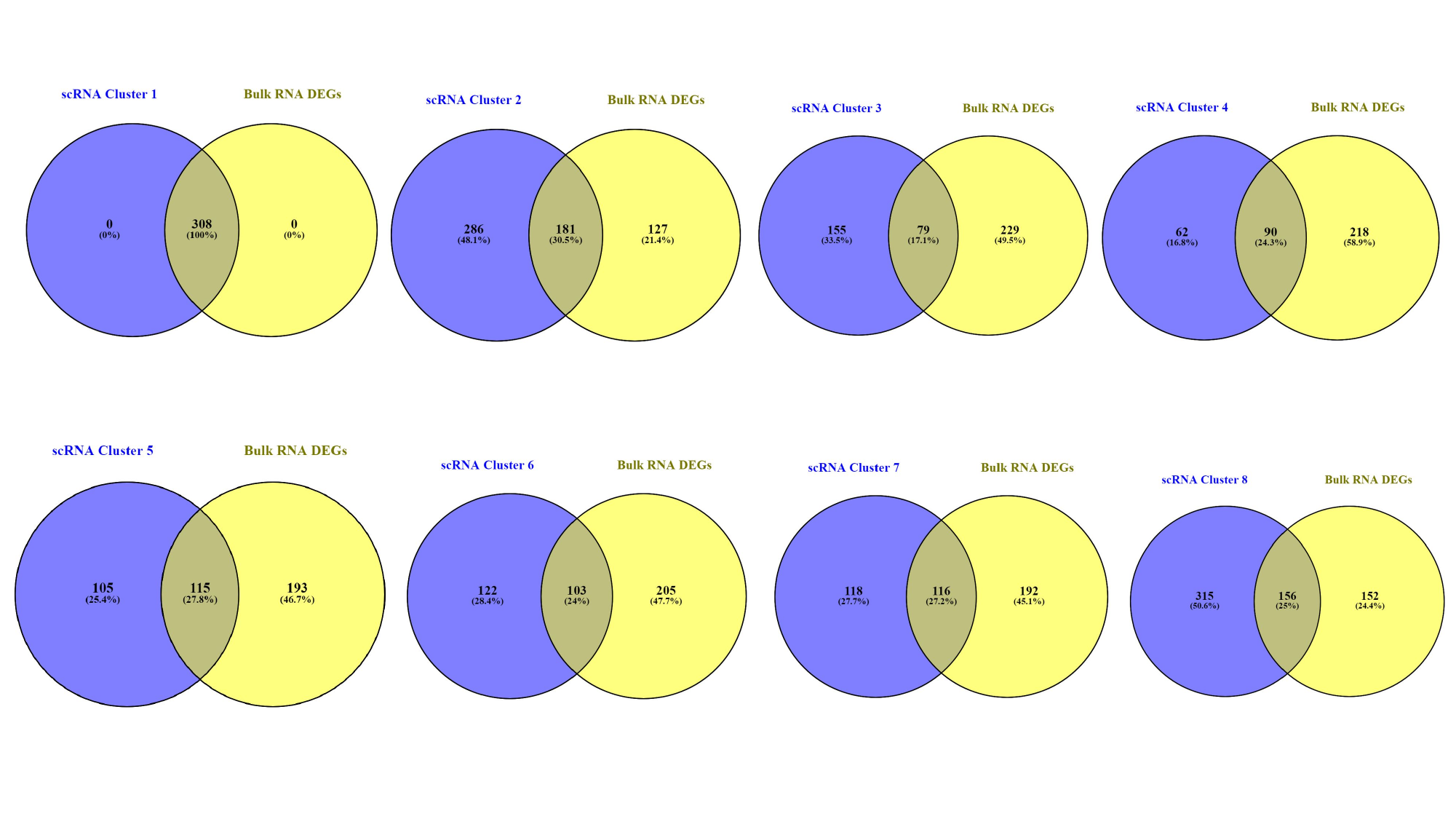

Supplement: Supplementary file 2 — Additional file 2: Figure 2: Area under the curve using logistic regression on the selected genes across all the datasets. [file 12967_2024_5414_MOESM2_ESM.pptx]

## Slide 1
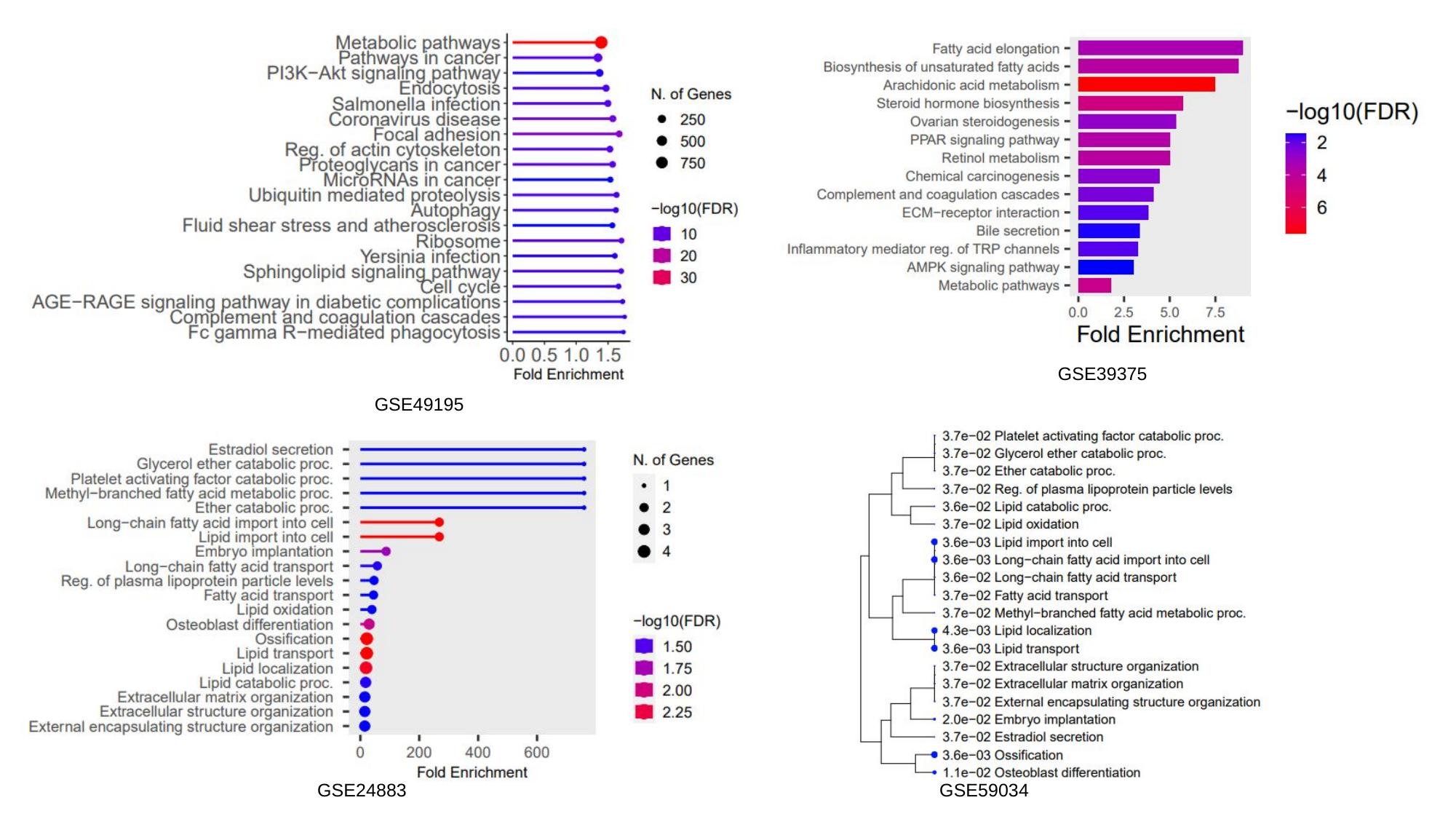

GSE39375
GSE49195
GSE24883 GSE59034

Supplement: Supplementary file 3 — Additional file 3: Figure 3: Pathway analysis of the genes identified from the data sets. Figure 3: Validation of identified genes of interest in human tissue. Plots of differentially expressed genes from computational analysis which were validated using qPCR, where each dot represents an individual patient, error bars represent standard error of the mean and p-value was calculated using unpaired student’s t-test. NW - lean and OB – obese. [file 12967_2024_5414_MOESM3_ESM.pptx]

## Slide 1
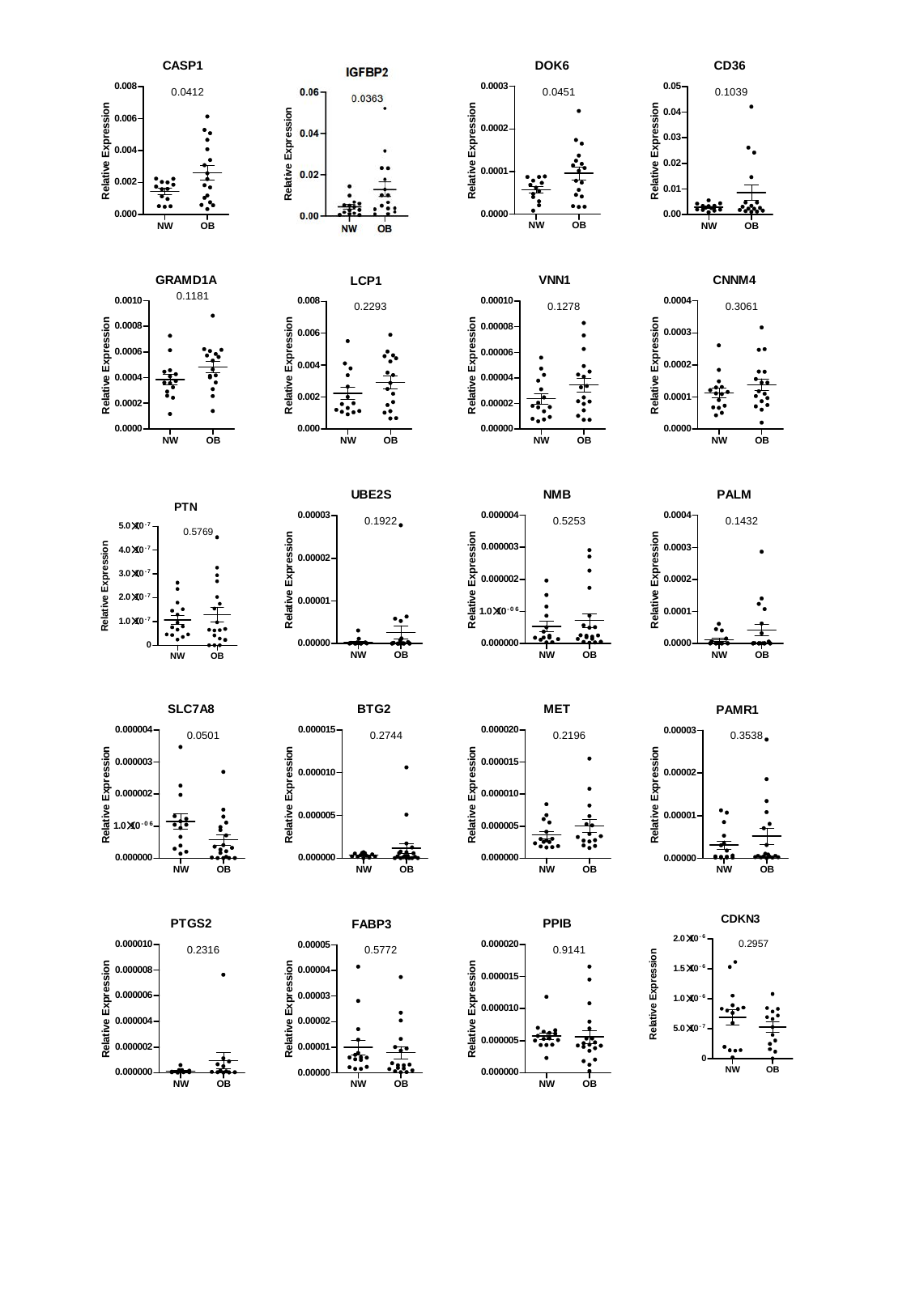

Supplement: Supplementary file 4 — Additional file 4: Figure 4: Validation of identified genes of interest in human tissue. Plots of differentially expressed genes from computational analysis which were validated using qPCR, where each dot represents an individual patient, error bars represent standard error of the mean and p-value was calculated using unpaired student’s t-test. [file 12967_2024_5414_MOESM4_ESM.pptx]
