# Supplementary material for: Cross-species transcriptomics identifies obesity associated genes between human and mouse studies
Source: J Transl Med. 2024 Jun 25;22:592. doi: 10.1186/s12967-024-05414-1 (PMC11197204; doi:10.1186/s12967-024-05414-1)
Supplement: Supplementary file 5 — Additional file 5: Table 1: List of primers and assay reagents used for qPCR validations. [file 12967_2024_5414_MOESM5_ESM.docx]

Supplementary Table 1. List of primers and assay reagents used for qPCR validations.

| Gene Symbol | Forward | Reverse | Assay ID | Dye Label | Assay | qPCR Assay |
| --- | --- | --- | --- | --- | --- | --- |
| **18S** | - | - | Hs99999901_s1 | FAM-MGB | TaqMan™ Gene Expression Assay (FAM) | Bio Rad iTaq™ Universal Probes One-Step Kit |
| **18S** | GTAACCCGTTGAACCCCATT | CCATCCAATCGGTAGTAGCG | VC00027N | SYBR | Merck Easy Oligos | Bio Rad iTaq™ Universal SYBR® Green One-Step Kit |
| **BTG2** | GGGAAGGGAACCGACATGCT | GAGCCCTTGGACGGCTTTTC | VC00027N | SYBR | Merck Easy Oligos | Bio Rad iTaq™ Universal SYBR® Green One-Step Kit |
| **CASP1** | - | - | Hs00354836_m1 | FAM-MGB | TaqMan™ Gene Expression Assay (FAM) | Bio Rad iTaq™ Universal Probes One-Step Kit |
| **CD36** | - | - | Hs00354519_m1 | FAM-MGB | TaqMan™ Gene Expression Assay (FAM) | Bio Rad iTaq™ Universal Probes One-Step Kit |
| **CDKN3** | CGGTTTATGTGCTCTTCCAGGT | TGACAGTTCCCCTCTGGTGC | VC00027N | SYBR | Merck Easy Oligos | Bio Rad iTaq™ Universal SYBR® Green One-Step Kit |
| **CNNM4** | - | - | Hs00950709_m1 | FAM-MGB | TaqMan™ Gene Expression Assay (FAM) | Bio Rad iTaq™ Universal Probes One-Step Kit |
| **DOK6** | - | - | Hs00913577_m1 | FAM-MGB | TaqMan™ Gene Expression Assay (FAM) | Bio Rad iTaq™ Universal Probes One-Step Kit |
| **FABP3** | GACGGGCAAGAGACCACACT | ATGTGGTGCTGAGTCGAGGG | VC00027N | SYBR | Merck Easy Oligos | Bio Rad iTaq™ Universal SYBR® Green One-Step Kit |
| **GAPDH** | - | - | Hs02786624_g1 | FAM-MGB | TaqMan™ Gene Expression Assay (FAM) | Bio Rad iTaq™ Universal Probes One-Step Kit |
| **GAPDH** | GGAGCGAGATCCCTCCAAAAT | GGCTGTTGTCATACTTCTCATGG | VC00027N | SYBR | Merck Easy Oligos | Bio Rad iTaq™ Universal SYBR® Green One-Step Kit |
| **GRAMD1A** | - | - | Hs00385151_m1 | FAM-MGB | TaqMan™ Gene Expression Assay (FAM) | Bio Rad iTaq™ Universal Probes One-Step Kit |
| **IGFBP2** | - | - | Hs01040719_m1 | FAM-MGB | TaqMan™ Gene Expression Assay (FAM) | Bio Rad iTaq™ Universal Probes One-Step Kit |
| **LCP1** | - | - | Hs00158701_m1 | FAM-MGB | TaqMan™ Gene Expression Assay (FAM) | Bio Rad iTaq™ Universal Probes One-Step Kit |
| **MET** | TGCCCGAAGTGTAAGCCCAA | TGCACTTGTCGGCATGAACC | VC00027N | SYBR | Merck Easy Oligos | Bio Rad iTaq™ Universal SYBR® Green One-Step Kit |
| **NMB** | ACTCGCGAGGCAACCTCTG | TGGATTTGGGGTGCGGGG | VC00027N | SYBR | Merck Easy Oligos | Bio Rad iTaq™ Universal SYBR® Green One-Step Kit |
| **PALM** | GGGAGATGGAGGTCCTGGC | TGCCTTGGACTTCAGGTGCT | VC00027N | SYBR | Merck Easy Oligos | Bio Rad iTaq™ Universal SYBR® Green One-Step Kit |
| **PAMR1** | GATGGAGCTGGGTTGCTGGA | ATTCCACTCTGCTCCAGGGC | VC00027N | SYBR | Merck Easy Oligos | Bio Rad iTaq™ Universal SYBR® Green One-Step Kit |
| **PPIB** | TGCGGCCGATGAGAAGAAGA | GACCAAAGATCACCCGGCCT | VC00027N | SYBR | Merck Easy Oligos | Bio Rad iTaq™ Universal SYBR® Green One-Step Kit |
| **PTGS2** | AGACGCCCTCAGACAGCAAA | TCCTGTCCGGGTACAATCGC | VC00027N | SYBR | Merck Easy Oligos | Bio Rad iTaq™ Universal SYBR® Green One-Step Kit |
| **PTN** | CCAAAGTGGAGAGAGGGGAAGAA | TGGTACTGTTGAGCCTGCAT | VC00027N | SYBR | Merck Easy Oligos | Bio Rad iTaq™ Universal SYBR® Green One-Step Kit |
| **SLC7A8** | CAGGCACCGAAACAACACCG | AGCACTCCCTTTGGCGAGAC | VC00027N | SYBR | Merck Easy Oligos | Bio Rad iTaq™ Universal SYBR® Green One-Step Kit |
| **UBE2S** | CGAGATCTGCGTCAACGTGC | ATCTCTGTGAGCAGACGGGC | VC00027N | SYBR | Merck Easy Oligos | Bio Rad iTaq™ Universal SYBR® Green One-Step Kit |
| **VNN1** | - | - | Hs01546812_m1 | FAM-MGB | TaqMan™ Gene Expression Assay (FAM) | Bio Rad iTaq™ Universal Probes One-Step Kit |
