# Supplementary material for: Cross-species transcriptomics identifies obesity associated genes between human and mouse studies
Source: J Transl Med. 2024 Jun 25;22:592. doi: 10.1186/s12967-024-05414-1 (PMC11197204; doi:10.1186/s12967-024-05414-1)
Supplement: Supplementary file 6 — Additional file 6: Table 2: Summary of the differentially expressed genes (DEG) identified in expression datasets analysed. [file 12967_2024_5414_MOESM6_ESM.docx]

Supplementary Table 2: Summary of the differentially expressed genes (DEG) identified in RNA-sequencing datasets analysed.

| **Datasets** | **Total DEG** | **adjPvalue (BH)** |
| --- | --- | --- |
| GSE24883 | 12 | <0.05 |
| GSE59034 | 7128 | <0.05 |
| GSE49195 | 13581 | <0.05 |
| GSE39375 | 12 | <0.2 |
| GSE219027 | 416 | <0.05 |
